# RETRACTION: A Randomized, Double‐Blind, Controlled Study Evaluating the Effects of Two Facial Serums on Skin Aging

**DOI:** 10.1111/srt.70333

**Published:** 2026-01-23

**RETRACTION**: D. Banov, M. Carvalho, S. Schwartz, and R. Frumento, “A Randomized, Double‐Blind, Controlled Study Evaluating the Effects of Two Facial Serums on Skin Aging,” *Skin Research and Technology* 29, no. 11 (2023): e13522, https://doi.org/10.1111/srt.13522.

The above article, published online on 23 November 2023 in Wiley Online Library (wileyonlinelibrary.com), has been retracted by agreement between *Skin Research and Technology*; and John Wiley & Sons A/S. Published by John Wiley & Sons Ltd. The article was accepted for publication following an insufficient peer review process that does not meet the standards of the journal's peer review policy. Post‐publication review revealed that the study lacked the formal ethical approval required by U.S. regulations for the type of research presented in the article. Additionally, the original study data cannot be made available for independent verification due to closure of the laboratory where the research was conducted.

While some co‐authors believed formal Institutional Review Board review was not required for this non‐therapeutic cosmetic study, the U.S. federal regulations (45 CFR 46) do not exempt this type of study from the requirement to obtain ethical approval. Despite not having this approval for the study, the Professional Compounding Centers of America (PCCA) emphasizes that the research was conducted ethically and without any misconduct or deception.

This retraction is therefore issued as administrative due diligence to correct the scholarly record and alert readers to these deficiencies in the publication process. It does not constitute a finding of research misconduct. The corresponding author was notified of this retraction and provided the opportunity to respond.

